# Executable Network Models of Integrated Multiomics Data

**DOI:** 10.1021/acs.jproteome.2c00730

**Published:** 2023-03-31

**Authors:** Mukta G. Palshikar, Xiaojun Min, Alexander Crystal, Jiayue Meng, Shannon P. Hilchey, Martin S. Zand, Juilee Thakar

**Affiliations:** † Biophysics, Structural and Computational Biology Program, University of Rochester Medical Center, Rochester, New York 14642, United States; ‡ University of Rochester, Rochester, New York 14627, United States; ∥ Department of Medicine, Division of Nephrology, University of Rochester Medical Center, Rochester, New York 14642, United States; ⊥ Clinical and Translational Science Institute, University of Rochester Medical Center, Rochester, New York 14642, United States; # Department of Microbiology and Immunology, University of Rochester Medical Center, Rochester, New York 14642, United States; ¶ Department of Biostatistics and Computational Biology, University of Rochester Medical Center, Rochester, New York 14642, United States

**Keywords:** B cells, hypoxia, cyclosporine, chemotaxis, multiomics, proteomics, Boolean networks, pathway analysis

## Abstract

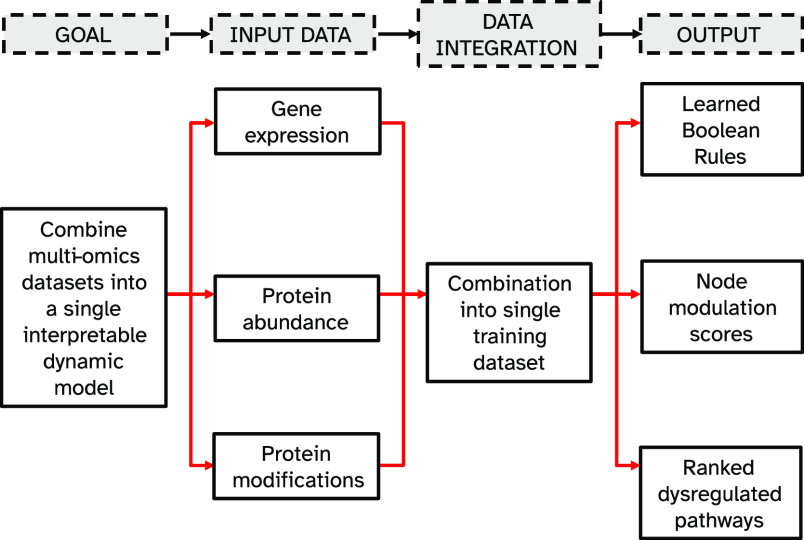

Multiomics profiling provides a holistic picture of a condition being examined and captures the complexity of signaling events, beginning from the original cause (environmental or genetic), to downstream functional changes at multiple molecular layers. Pathway enrichment analysis has been used with multiomics data sets to characterize signaling mechanisms. However, technical and biological variability between these layered data limit an integrative computational analyses. We present a Boolean network-based method, multiomics Boolean Omics Network Invariant-Time Analysis (mBONITA), to integrate omics data sets that quantify multiple molecular layers. mBONITA utilizes prior knowledge networks to perform topology-based pathway analysis. In addition, mBONITA identifies genes that are consistently modulated across molecular measurements by combining observed fold-changes and variance, with a measure of node (i.e., gene or protein) influence over signaling, and a measure of the strength of evidence for that gene across data sets. We used mBONITA to integrate multiomics data sets from RAMOS B cells treated with the immunosuppressant drug cyclosporine A under varying O_2_ tensions to identify pathways involved in hypoxia-mediated chemotaxis. We compare mBONITA’s performance with 6 other pathway analysis methods designed for multiomics data and show that mBONITA identifies a set of pathways with evidence of modulation across all omics layers. mBONITA is freely available at https://github.com/Thakar-Lab/mBONITA.

## 1 Introduction

The etiology of complex host responses to diseases involves changes at multiple layers of molecular regulation, including the genomic, transcriptional, post-transcriptional, and metabolic levels. These molecular levels can be profiled by generating “omics” data sets such as transcriptomics (mRNA levels), proteomics (protein levels), and phosphoproteomics (phosphoprotein levels), which are individually extraordinarily rich and allow sophisticated inferences about molecular signaling events and clinical observations.^1^ The number and complexity of these data sets has steadily increased.^2−4^ Strategies for the interpretation of these complex data sets are designed to tease apart the underlying changes in molecular signaling, and to integrate the data into an interpretable low-dimensional representation.^5,6^). In particular, pathway enrichment analysis allows the identification of modulated biological processes by two main classes of methods:^7,8^ overrepresentation analysis and functional class scoring, and topology-based pathway enrichment analysis. However, technical and biological variability between these layered data sets present challenges for integrative computational analyses in general and pathway analysis in particular.

Discrete-state network modeling characterizes regulatory interactions in networks with Boolean rules or gates that define signal flow through the network.^9^ These network models are part of a larger class of *executable* models, which can be simulated to investigate the behavior of a dynamic system, in this case biological signaling networks.^10^ Discrete-state network models can be simulated either synchronously or asynchronously to identify limit cycles or attractors that correspond to network-specific states/phenotypes.

We have recently published two algorithms that infer regulatory rules for prior knowledge networks (PKNs) from omics data, i.e., to generate executable network models.^11,12^ These inferred regulatory rules are used to simulate *in silico* perturbation to calculate the influence of nodes over signaling through the network. These perturbation-based scores are combined with expression data to perform pathway analysis. However, both these methods rely on information from a single omics training data set to perform rule inference and pathway analysis.

Here, we present a method, multiomics Boolean Omics Network Invariant Time Analysis (mBONITA), to (**1**) use multiple layers of omics data to improve inference of regulatory rules and reconstruct executable network models, (2) use the abundance levels from all layers to calculate node importance scores, and (**3**) perform pathway analysis that incorporates information from multiple omics data sets. We demonstrate the utility of this algorithm on our multiomics data set from RAMOS B cells grown under hypoxic conditions and treated with cyclosporine A (CyA). CyA modulates O_2_ -dependent chemotaxis in human B cells via the transcription factor HIF1α. Transcriptomics, proteomics, and phosphoproteomics were measured because several downstream signaling cascades are post-transcriptionally regulated upon HIF1α activation. Previous analysis of proteomic and phosphoproteomic levels shows modulation of cytoskeletal rearrangement, which was also experimentally validated.^13,14^ Here we present the first description of the analysis of transcriptomic data from this experiment, and the first description of the integration of the three data sets (transcriptomics, proteomics, phosphoproteomics) from this system.

Our method can effectively use this multiomics data set in combination with PKNs from KEGG^15^ and WikiPathways^16,17^ to improve the inference of regulatory rule sets for PKNs, thus increasing the reliability of the executable models. We used these improved rule sets to calculate gene/node modulation scores that incorporate all available expression information and the network topology. We then used mBONITA to identify pathways that are significantly modulated in the three contrasts, including pathways that are not significantly modulated in individual data sets. Here, “contrasts” refers in general to the comparisons of experimental conditions that are considered when identifying significantly modulated pathways or gene sets. In the case of this experiment, we considered three contrasts (or comparisons) for pathway analysis: (**1**) 19% O_2_, CyA– vs 1% O_2_, CyA–, (**2**) 1% O_2_, CyA+ vs 1% O_2_, CyA–, and (**3**) 19% O_2_, CyA– vs 1% O_2_, CyA+. We compare these pathways to those identified by the other pathway analysis methods PaintOmics,^18,19^ CAMERA^20^ in combination with Fisher’s method of *p*-value combination as suggested in ReactomeGSA,^21^ LeapR,^22^ multiGSEA,^23^ and ActivePathways,^24^ and show that mBONITA identifies the most relevant pathways to these conditions. Furthermore, we use mBONITA to calculate node modulation scores for a custom large signaling network describing the HIF1α-mediated signaling in B cells and show that it is highly modulated across three contrasts. We show that the genes identified by mBONITA are not identified by differential expression analysis alone and contain strong candidates for experimental validation such as DIAPH3 (Diaphanous Related Formin 3), a member of the formin family that is involved in actin remodeling and regulation of cell movement and adhesion,^25^ as well as ACTR2 (Actin Related Protein 2), which is a component of the ARP2/3 complex located at the cell surface, is involved in modulating cell shape and motility through actin assembly, and is important for spatial patterning of the B cell immune synapse formation.^26^

It is important to note that mBONITA can be applied to cross-sectional or “snapshot” data for regulatory rule inference, unlike most other dynamic modeling techniques, which require time-course data to learn models. mBONITA can identify highly modulated genes, and can perform pathway analysis using multiple sources of high-throughput molecular measurements to present a complete picture of modulated signaling. For example, mBONITA identified a paired modulation of cellular motility and glucose metabolism under hypoxic conditions in B cells. Thus, mBONITA can be used to perform integrative analysis using multiple omics data sets.

## 2 Materials and Methods

### 2.1 Transcriptomics Data Collection and Analysis

RAMOS cells were maintained in a 37 °C, 5% CO_2_, humidified incubator in cR10 media (RPMI 1640 media supplemented with 10% heat inactivated fetal bovine serum (FBS), 50 U/mL Penicillin, 50 μg/mL Streptomycin, and 50 μM 2-Mercaptoethanol). RAMOS cells, in triplicate, were treated with either 0 or 1 μg/mL cyclosporine A (CyA) and incubated at either 19% O_2_ (traditional tissue culture) or 1% O_2_ for 24 h. After incubation with CyA at the indicated O_2_ conditions, cells were harvested by centrifugation and washed 3× with phosphate buffered saline (PBS). RNA was extracted from the resultant cell pellets using TRIzol Plus RNA Purification Kits according to the manufacturer’s recommendations (Invitrogen). Single-end RNA-sequencing was performed on the Illumina NextSeq 550. Raw data were formatted using bcltofastq-2.19.0. Sequence reads were trimmed for adaptor sequence/low-quality sequence using Trimmomatic-0.36.^27^ Trimmed sequence reads were mapped to Reference Genome hg38/GencodeV28 using STAR_2.6.0c.^28^ Read quantification was performed using featureCounts from the R package subread version 1.34.7^29^ using genome assembly GRCh38.p12.

Differentially expressed (DE) genes were identified using DESeq2.^30^ The R package ashr was used for log fold change shrinkage.^31^ Genes with a Benjamini–Hochberg adjusted *p* < 0.05 and an absolute log_2_-fold change >0.5 were identified as being DE. Heatmaps were prepared using ComplexHeatmap.^32^ Overrepresentation analysis of DE genes was performed with the R package clusterprofiler, using gene sets of canonical KEGG pathways from the MSigDB database.^15,33,34^ Gene sets were identified as being overrepresented if the unadjusted *p* < 0.05.

#### Phosphoproteomics and Phosphoproteomics Data Acquisition

Here, we present brief summaries of sample preparation, data acquisition and processing for the proteomics and phosphoproteomics used in our analysis. These experiments are completely described in our previous publications, where these data sets were first described.^13,14^

#### Phosphoproteomics Data Acquisition

RAMOS B cells were cultured at either 19% or 1% O_2_ and treated with CyA, flash-frozen in liquid nitrogen and stored at −80 °C until protein sample preparation was performed, as previously described.^14^ Phosphopeptide enrichment was performed based on a modified version of a previously published titanium dioxide bead-based protocol.^35^ The enriched phosphopeptides were isobarically labeled using Ten-plex Tandem Mass Tag (TMT) reagents (Thermo Fisher Scientific, Rockford, IL, USA). Labeled phosphopeptides were dried, reconstituted, eluted from conditioned reversed-phase fractionation spin columns (Thermo Fisher Scientific, Rockford, IL, USA) as described. Eluted fractions were injected in triplicate for LC–MS analysis. The LC–MS system consists of a Dionex Ultimate 3000 nano LC system, a Dionex Ultimate 3000 gradient micro LC system with a WPS-3000 autosampler, and an Orbitrap Fusion Lumos mass spectrometer (Thermo Fisher Scientific, San Jose, CA, USA).^14^ MS acquisition was operated using the Synchronous Precursor Selection (SPS)-MS3 method.^36^ Data were processed using Proteome Discoverer 2.2 (Thermo Fisher Scientific, San Jose, CA, USA) and peptides were identified with Sequest HT using Swiss-Prot and validated by Percolator. Phosphosites were localized by ptmRS.^14^ Detailed parameters are listed in For further analysis, phosphopeptides were mapped to corresponding proteins/genes and the phosphopeptide with the highest abundance was considered as a representation of the abundance of the protein. The normalized abundance for undetected phosphopeptides was imputed to 0. We considered 11,652 phosphopeptides mapping to 3037 proteins for downstream analysis.

#### Proteomics Data Acquisition

RAMOS B cells were cultured, treated, harvested, lysed, and digested as described previously.^13^ Samples were tagged with tandem mass tag (TMT) ten-plex reagents (0.2 mg) (Thermo Fisher Scientific, Waltham, MA) and fractionated as described. Peptides were injected onto a 30 cm C18 column packed with 1.8 μm beads (Sepax), with an Easy nLC-1000 HPLC (Thermo Fisher Scientific, Waltham, MA), connected to a Q Exactive Plus mass spectrometer (Thermo Fisher Scientific, Waltham, MA) and data were acquired as described previously. Peptides were identified using SEQUEST and Swiss-Prot database within the Proteome Discoverer software platform, version 2.2 (Thermo Fisher Scientific, Waltham, MA). Parameters were selected as described previously.^14^ Protein abundances were calculated by summing the intensities of the reporter ions from each identified peptide, while excluding any peptides with an isolation interference of 30% or more. Low abundance proteins with less than one count per experiment/replicate were removed resulting in 5048 proteins.

### 2.2 Data Processing for Pathway Analysis

Proteomics and phosphoproteomics data were collected and processed as described.^13,14^ We retained only samples from the experimental conditions represented in all three data sets (Supplementary Table S1). In the case of the proteomics and phosphoproteomics data sets, we mapped protein names to gene names using Entrez and retained these gene names for downstream analysis, for consistency between data sets.

Phosphopeptides were mapped to corresponding proteins/genes. Proteins were often mapped to multiple phosphopeptides. We assumed that of these multiple phosphopeptides, only the phosphopeptide with the highest abundance represents the most relevant phosphorylated species. This decision was made because of the confidence in the measurement of the phosphorylated species. In addition, this decision simplified the mapping of individual protein species to the nodes in the prior knowledge networks, which typically do not contain nodes corresponding to multiple phosphorylated versions of a protein (i.e., there is only node per protein/gene).

While we did consider imputing phosphoproteomic events on the basis of multiple measured phosphoproteomic species available in our data set, which may have provided more insights into phosphorylation processes, this may have also introduced a number of false nodes and edges due to the technical limitations of the phosphoproteomic assay. Specifically, we were concerned that imputing phosphoproteomic events on the basis of measured phosphoproteomic species would result in the addition of phosphoproteomic species that were not true representations of the species but were instead technical errors or partial peptides that did not represent the true phosphorylation status of the protein. The major caveat of this procedure is that multiple phosphorylation species are not represented in the final network models, and there is a corresponding loss of information on signal flow. We note, however, that the extension of mBONITA to include these species is trivial and much depends on the availability of reliable PKNs that contain information on phosphorylation events and their accurate measurements.

In the case of both the proteomics and phosphoproteomics data sets, we discarded observations for genes whose median value was 0. Processed proteomics and phosphoproteomics data were log_2_(*x* + 1)-transformed and transcriptomics data were log_2_(RPM)-transformed.

### 2.3 Multiomics Network Modeling and Pathway Enrichment Analysis with mBONITA

**Figure 1:**
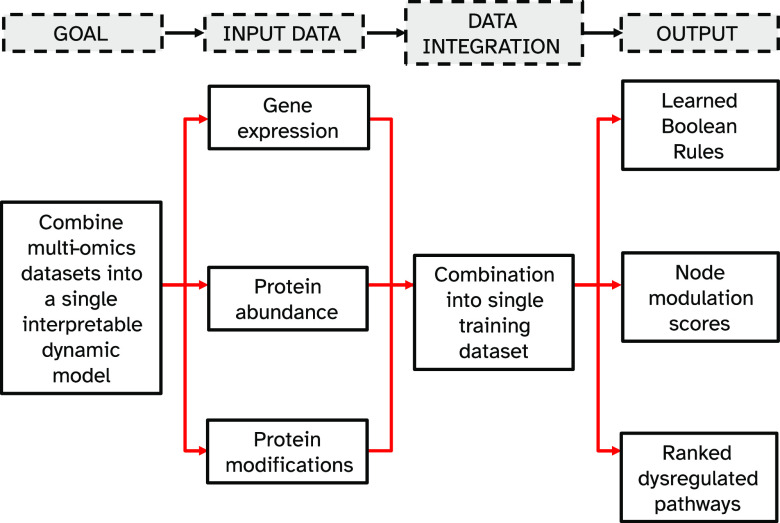
mBONITA integrates information from multiple omics data sets to learn a consensus set of logic rules for prior knowledge networks (PKNs), simulate and perturb PKNs *in silico*, calculate condition-specific node modulation scores, and perform topology-based pathway analysis.

Multiomics Boolean Omics Network Invariant-Time Analysis (mBONITA) extends our previous Boolean modeling and pathway analysis approaches.^11,12^mBONITA is a three-step process that requires four inputs (Figure 1): (**1**) prior knowledge networks in graphml format, defining the topology of the signaling network(s) , (**2**) a combined data set including gene/protein expression values from the multiomics data sets under consideration, (**3**) a design matrix specifying the treatment for each sample in the training data set, and (**4**) a contrast matrix describing comparisons of interest. mBONITA is tested for scenarios where conditions are matched across all omics data sets, although we can extend it to include unmatched data sets. In the first step, pathways are downloaded from KEGG using the KEGG API if desired, and prepared for rule inference.^37^ In the second step, mBONITA infers Boolean rules with a combination of a genetic algorithm and a local search as described previously.^12^ We have reimplemented the BONITA.^12^ Python tool in Python 3, resulting in significant upgrades in speed, and use this updated tool as a basis for the mBONITA module. In the third and final step, node importance scores (*I*_*g*_), which quantify the effect of individual nodes *g* over signal flow through a network, were calculated by *in silico* perturbation of networks as previously described.^12^

The importance score *I*_*g*_ for a gene *g* is given by the difference between the steady states/attractors of the network (which correspond to network-specific phenotypes) identified under the two perturbation conditions. In other words, this score quantifies the difference between the effect on the network of a knock-in and a knockout of a given node.
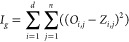
1In eq 1, *j* ranging from 1 to *n* indicates nodes in the network, *i* ranging from 1 to *d* indicates samples, *O*_*i*,*j*_ and *Z*_*i*,*j*_ are the steady states of nodes *j* in sample *i* when *g* = 0 (knockout) and *g* = 1 (knock-in) across all iterations, as calculated by mBONITA^12^ These scores are specific to network topology, not experimental condition. Node importance scores are weighted by the fold-changes for each contrast from each data set, the standard deviation of the gene across each data set, and the strength of the evidence for that gene across all data sets, to calculate a node modulation score (eq 3). Each gene in the pathway is assigned an evidence score from 1 to the total number of omics data sets under consideration using (eq 2). The evidence score *E*_*g*_ for a gene is given by
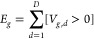
2where *D* is the number of multiomics data sets, and *V*_*g*,*d*_ is the measured abundance value of gene *g* in data set *d*. The modulation score for a gene *M*_*g*_ with importance score *I*_*g*_, in a particular contrast *C* and a contrast-specific fold change FC_*C*_, is given by
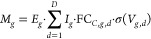
3A pathway modulation score is calculated by summing up the node modulation scores for nodes in the pathway (eq 4). The node and pathway modulation scores integrate the overall changes in the state of a protein and the overall system, respectively, across data types. The pathway modulation score *M*_*p*_ for a pathway *p* with *G* genes (or nodes) is given by
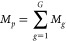
4*P*-values for the pathway analysis are calculated as follows. Importance scores that have been calculated for a specific network are weighted with randomly sampled, contrast-specific, fold changes and randomly sampled coefficients of variation (as in eq 2). These are summed to generate a random pathway modulation score (as in eq 3). This procedure is repeated 1000 times to generate a random distribution of pathway modulation scores. The real pathway modulation score is compared to this distribution and a *z*-score is calculated. *P*-values are calculated for each pathway (i.e., each pathway modulation score) using the cumulative distribution function of the standard normal distribution (*p*-value = 1 – ϕ(*z*-score)).

In a typical mBONITA analysis, these steps are automatically performed for all KEGG pathways that have at least 5 genes in the training data set. This parameter can be tuned as appropriate. The outputs of this analysis are a table of *p*-values for each pathway in each contrast, graphml files annotated with fold-changes and importance scores, ready to be imported into network visualization software such as Cytoscape^38^ or Gephi,^39^ and tables of node modulation scores for each combination of pathway and contrast.

We reimplemented our original BONITA^12^ pipeline in Python 3 for speed, and used it to infer Boolean rules for a combined data set comprising samples for conditions that were profiled in all three data sets (Supplementary Table S1). For each of these experiments, we used all KEGG networks with an overlap of 5 or more genes with the training data set. We also used the mBONITA pipeline to infer Boolean regulatory rules and node modulation scores for a custom network constructed by composing the KEGG networks in (Supplementary Table S2). These signaling networks are known to be involved in the response of B cells to hypoxia or are linked to chemotaxis.^13,14^ Dynamic modeling of such custom networks allows the investigation of cross-talk between multiple pathways.

### 2.4 Comparison of mBONITA to Other Multiomics Pathway Analysis Methods

We compared pathway analysis with mBONITA to five other pathway analysis methods which have been designed (or modified) to use multiomics data sets. Data were processed as described above. We used *Homo sapiens* pathways from KEGG as gene sets for all analyses to ensure consistency with the mBONITA analysis. We note that all of these methods can be used with gene sets from other sources. The differential expression analysis for all data sets was performed with limma,^40^ using the Reactome Pathway Browser web tool^21^ and used as input to multiGSEA.^23^ PaintOmics^18,19^ requires a list of relevant genes, which we defined as those with an absolute log_2_-foldchange ≥ the fourth quantile. *P*-values were combined with Stouffer’s method. We used the implementation of CAMERA^20^ in the limma R package^40^ and combined *p*-values using Stouffer’s method as implemented in the metap R package.^41^ We used the “enrichment-comparison” method implemented in leapR. The *p*-values calculated with limma (as above) were used as input to ActivePathways.^24^ For the genes/proteins only identified in some data sets, the *p*-value of differential expression was assigned to 1, as recommended in the ActivePathways documentation. Complete results from these methods are available as Supplementary Files (Supplementary File S4, Supplementary File S5, Supplementary File S6, Supplementary File S7, Supplementary File S8).

### 2.5 Data and Software Availability Statements

The transcriptomics data set described in this manuscript has been deposited to NCBI-GEO with the accession number GSE212853. The mass spectrometry phosphoproteomics and proteomics data sets are available at the ProteomeXchange Consortium partner repository PRIDE^42^ with the data set identifiers PXD036167 and PXD037004 respectively. Lists of differentially expressed genes/proteins and code to regenerate all figures in this manuscript, as well as the source code, documentation, and tutorials for the mBONITA pathway analysis module and the BONITA3 Python tool, are available at https://github.com/Thakar-Lab/mBONITA and https://github.com/Thakar-Lab/BONITA-Python3, respectively.

## 3 Results and Discussion

### 3.1 Integrative Analysis by mBONITA Improves Inference of Regulatory Mechanisms

**Figure 2:**
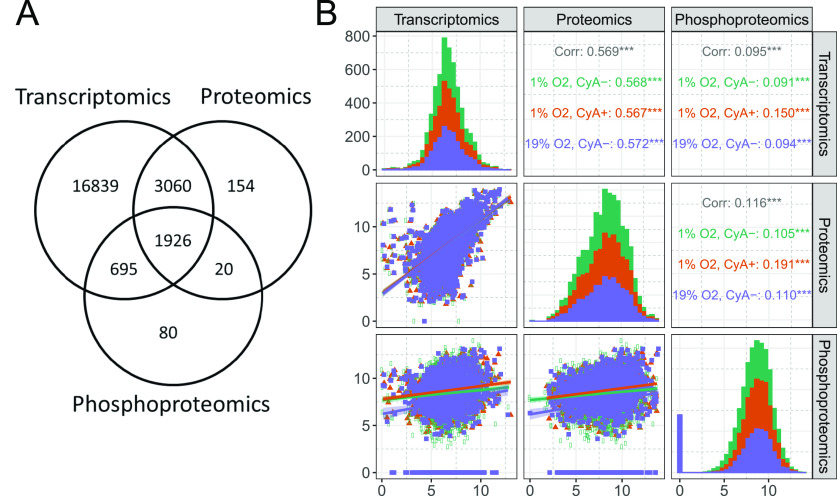
RAMOS B cells treated with cyclosporine A (CyA) and grown at different O_2_ tensions were profiled at three molecular layers. (A) 1926 genes were profiled in all three omics data sets (median expression >0). (B) Correlations across different omics data sets. Expression data were processed and log_2_-normalized as described in the Materials and Methods. Only genes profiled in all data sets were compared. Distinct experimental conditions are indicated by colors as shown in the figure annotations.

We used mBONITA to perform an integrative pathway analysis of three omics data sets generated from RAMOS B cells grown under hypoxic and normoxic conditions, in combination with the calcineurin inhibitor cyclosporine A (CyA) (Supplementary Table S1). Data sets were processed as described in the Materials and Methods. We considered only conditions that were profiled in all three data sets (Supplementary Table S1). Preliminary analysis showed that there were significant differences in the number of molecular entities profiled in the three data sets. 1926 genes were profiled in all three data sets out of a total of 22774 genes with nonzero abundance in at least one sample (Figure 2A). In addition, the measured abundances of these 1926 genes had low Spearman correlation across data sets even when separated by condition, ranging from 0.6 (transcriptomics and proteomics) to 0.1 (transcriptomics and phosphoproteomics), *p* < 0.01 (Figure 2B). A comparison of the differentially modulated pathways identified in the three data types showed that the pathways identified in the proteomic and phosphoproteomic analysis were not different at the transcriptomic level (Supplementary Figure S1A–C). Although theoverlap of significantly modulated pathways was low, the correlations between transcriptomics and proteomics abundance levels were high, suggesting that transcriptomics could contribute to learning regulatory mechanisms in an integrative analysis. mBONITA’s rule determination algorithm returns a set of candidate regulatory rules per node, which all equivalently describe the training data.^11,12^. We refer to these rules as the equivalent rule set or *ERS*. A larger *ERS* is observed when the algorithm is unable to distinguish between multiple types of regulation given the training data, i.e., the larger the size of the *ERS* for a gene, the lower the confidence of learning the regulatory information. The size of the *ERS* has clearly defined numerical limits. For example, there are 127 possible Boolean rules for a node with three upstream regulators (this is the most complex case considered by mBONITA). Here, we use the size of the *ERS* returned by mBONITA as a proxy for the uncertainty in rule determination.

**Figure 3:**
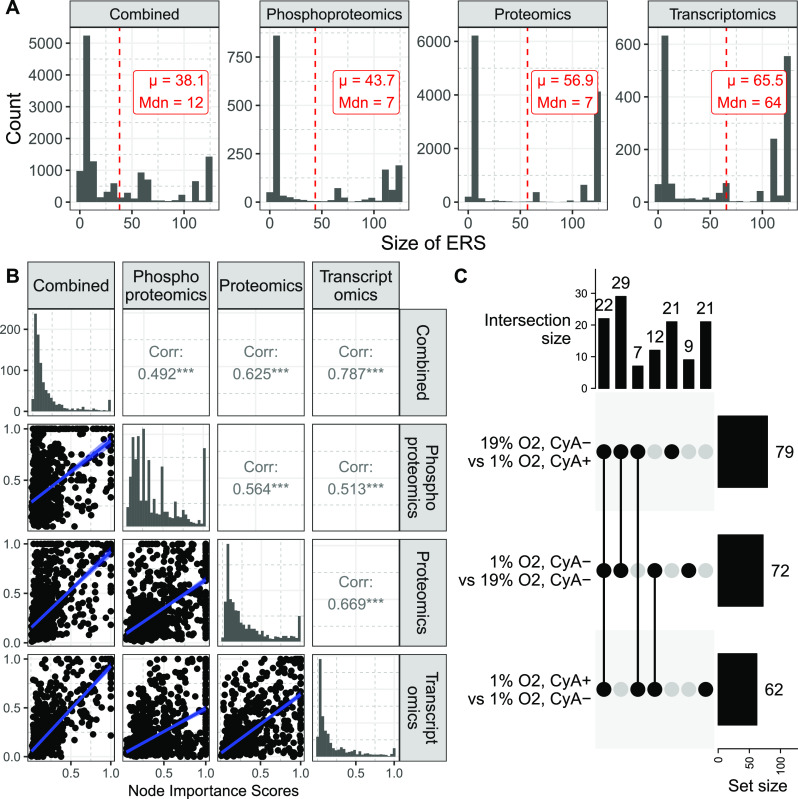
mBONITA identifies mechanisms of hypoxia-mediated chemotaxis from a multiomics data set from RAMOS B cells grown under three experimental conditions. All experiments used pathways downloaded from KEGG. (A) Inferred rule set sizes (*ERS*) for each omics data set and for the integrated analysis. Only nodes with in-degree ≥3 are shown. The mean (μ) and median (*Mdn*) of the ERS are shown for each data set. The red dashed line indicates μ. (B) Comparison of the mBONITA node importance scores learned from each experiment. *Corr* indicates the Pearson correlation coefficient. *** indicates that *p* < 0.01. (C) Number of differentially regulated (Benjamini–Hochberg corrected *p* < 0.05) KEGG pathways identified by mBONITA in the three contrasts.

While the transcriptomics data set was much larger than the proteomics data sets in terms of the number of genes profiled, the size of the *ERS* was significantly higher than when the algorithm was trained on the proteomics and phosphoproteomics data sets. This is consistent with previous observations suggesting oxygen levels modulate post-transcriptional and phosphorylation events, rather than simply transcriptomic events.^43−45^ Importantly, overall the *ERS* was higher for all three data types when they were individually used for rule inference. Notably, mBONITA’s rule inference algorithm inferred smaller (and hence more high-confidence) rule sets when all three omics data sets were used together for training (Figure 3A, *t* test, *p* < 0.01).

### 3.2 mBONITA Identifies Mechanisms of Hypoxia-Mediated Chemotaxis in RAMOS B Cells

As shown above, the integrative analysis by mBONITA significantly improved the inference of regulatory rules (Figure 3A). Next, we wanted to evaluate the node importance score calculated by mBONITA. The node importance score quantifies the influence of individual nodes over signal flow through the network. These scores were overall positively correlated (Pearson correlation coefficient >0.49, *p* < 0.01) across data types and when the rules were inferred using a combined data set (Figure 3B). The importance score correlation was higher for proteomics and transcriptomic data sets. The importance scores calculated using combined omics data were highly correlated to those obtained using transcriptomics data, indicating that the transcriptomics data set had a strong influence on the calculation of these scores. We note that node importance scores are dependent solely on network topology and inferred Boolean rules and are independent of data set-specific fold change. Simulation experiments in which network topologies were shuffled to elucidate this relationship (Supplementary Note S1) indicated that mBONITA’s node importance score can identify highly influential nodes with a high-degree of confidence but cannot distinguish between nodes with, low influences. These observations underscore the augmentation in signaling information that can be obtained from multiple molecular layers.

We then performed pathway analysis with mBONITA (eq 4) on the combined omics data sets and identified pathways modulated in three contrasts (Figure 3C, Supplementary File S3). In line with experimental observations that treatment with CyA under varying oxygen tensions modulates cellular chemotaxis,^13,14^mBONITA identified multiple cytoskeletal-related modulated pathways across the three contrasts. The regulation of actin cytoskeleton and glucagon signaling pathways are dysregulated between 19% O_2_ CyA– samples and 1% O_2_ CyA– samples, indicating a paired modulation of cellular motility and glucose metabolism under hypoxic conditions. The glucagon signaling and axon guidance pathways were also modulated between 19% O_2_ CyA– and 1% O_2_ CyA+ samples. The herpes simplex virus 1 infection pathway, which contains many genes linked to cytoskeletal remodeling, was similarly modulated between 1% O_2_ CyA+ and 1% O_2_ CyA– samples. Crucially, the HIF1α signaling pathway is modulated only between 1% O_2_, CyA– and 1% O_2_, CyA+ samples, indicating that treatment with CyA is responsible for the differences in HIF1α-mediated phenotypic effects such as altered chemotaxis. Indeed, we have previously shown in our proteomic and phosphoproteomics data sets that HIF1α-regulated proteins and cytoskeletal pathways are modulated by O_2_ levels.^13,14^ This small list of pathways identified by mBONITA is highly interpretable and specific to the condition under study, especially in comparison to currently available methods for multiomics pathway analysis, as shown in Section 3.4 and Figure 5.

### 3.3 Pathway-Based Prioritization of Genes in a Signaling Network with mBONITA

**Figure 4:**
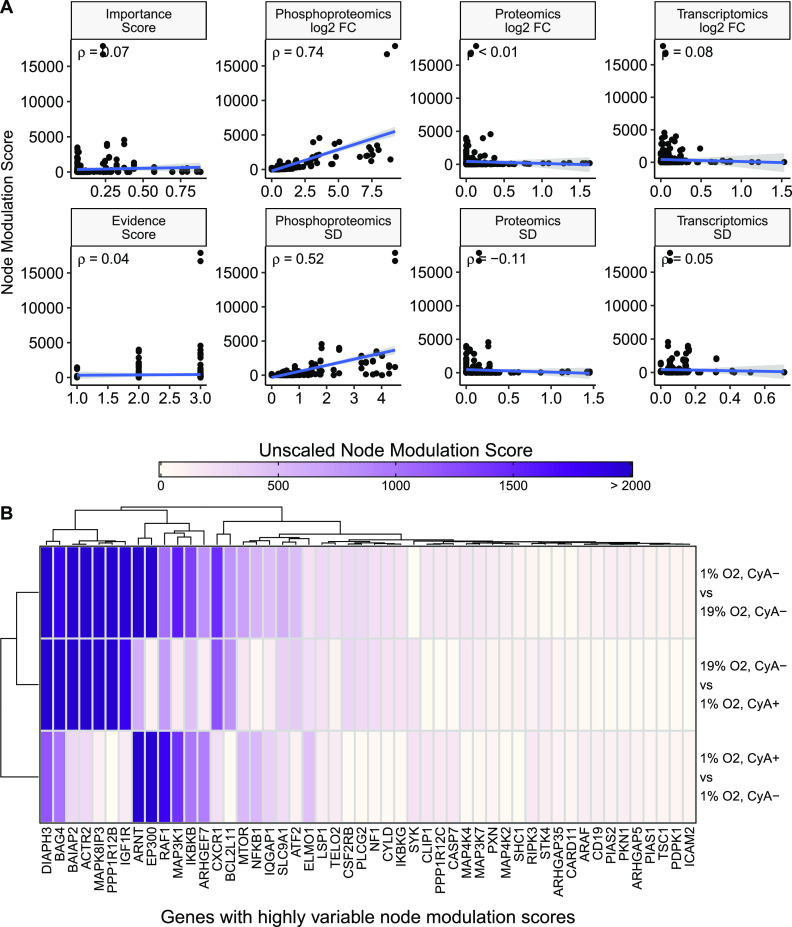
Pathway-based prioritization of genes in a LSP1/HIF1A-centric signaling network with mBONITA. (A) Correlation between calculated node modulation scores *N*_*m*_ and its individual components (eq 3). ρ indicates the Pearson correlation coefficient (*p* < 0.01 in all cases). log_2_FC = log_2_ fold-change and SD = standard deviation. (B) The 50 nodes with highest variation in *N*_*m*_ across the three contrasts. Values above 2000 are grouped and indicated as >2000 on the color bar.

mBONITA calculates a node modulation score that incorporates a measure of node influence over a signaling network, observed contrast-specific variation, and an evidence score measuring the presence of that protein given the provided multiomics data (eq 2, eq 3). We propose that this score identifies influential nodes that are both highly variable and highly influential in signaling and hence are good candidates for experimental validation. We demonstrate the effectiveness and interpretability of mBONITA’s node modulation score (*N*_*m*_) on a previously described custom network describing the HIF1α-mediated response of B cells to hypoxia and treatment with CyA.^14^ The individual components of *N*_*m*_ are not highly correlated with the overall *N*_*m*_ with the exception of fold-changes from the phosphoproteomics data set (Pearson correlation coefficient = 0.74, *p* < 0.01) (Figure 4A). However, no component of *N*_*m*_ clearly drives its magnitude, which ranges from close to 0 to 18000 in this network. Examination of the nodes with the most variable *N*_*m*_ identifies genes linked to cytoskeletal rearrangement and the hypoxia response. DIAPH3 is involved in actin polymerization and stabilization of microtubules during cytokinesis.^46−48^ BAIAP2 is an insulin receptor tyrosine kinase substrate which is involved in CDC42-mediated actin cytoskeletal remodeling.^49−51^ ACTR2 is a component of the Arp2/3 complex, which mediates actin cytoskeletal assembly and is required for cell motility.^52^ ARNT (HIF1β) is a cofactor of HIF1α and has been shown to be modulated under hypoxic conditions.^53−55^ These genes were not identified in either differential expression analyses^13,14^ (Supplementary Figure S1) or by graph-theoretic analyses of the signaling network (data not shown) (Figure 4B). They were also not identified when only the phosphoproteomics data were used with BONITA for a similar network.^14^

### 3.4 Comparison of mBONITA Performance with the Other Pathway Analysis Methods

**Figure 5:**
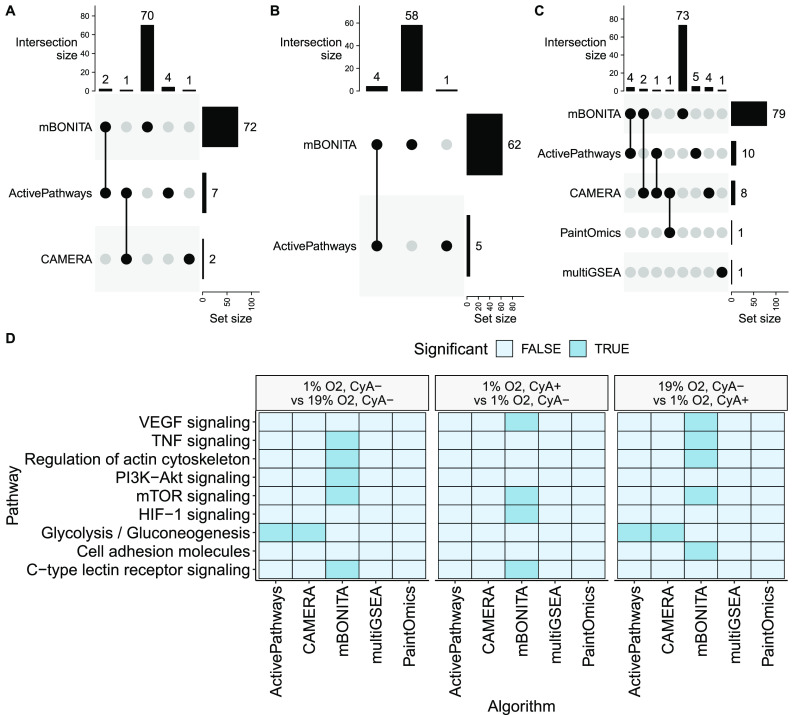
Comparison of mBONITA performance with the other pathway analysis methods. (A–C) Numbers of differentially regulated KEGG pathways identified from combination multiomics data in three contrasts: (A) 19% O_2_, CyA– vs 1% O_2_, CyA–. (B) 1% O_2_, CyA+ vs 1% O_2_, CyA–. (C) 19% O_2_, CyA– vs 1% O_2_, CyA+. (D) Significance of pathways known to be involved in the hypoxia-mediated response to CyA, for all three contrasts. Only pathways identified as significant from a combined data set by at least one method are shown. Pathways are defined as significantly modulated if the Benjamini–Hochberg corrected *p* < 0.05.

We compared mBONITA to five other pathway analysis methods designed for multiomics data sets, ActivePathways,^24^ CAMERA^20^ in combination with Stouffer’s method of *p*-value combination as suggested by the authors of ReactomeGSA,^21^ PaintOmics4,^18,19^ leapR,^22^ and multiGSEA^23^ (Figure 5D). Complete pathway analysis results with each of these methods are presented in the Supplementary Data (Supplementary File S4, Supplementary File S5, Supplementary File S6, Supplementary File S7, Supplementary File S8). Across all three tested contrasts, mBONITA found the most significantly modulated pathways (Benjamini–Hochberg adjusted *p* < 0.05) (Figure 4A–C), most of which were exclusively identified by mBONITA. LeapR did not identify modulated pathways in any contrast. ActivePathways consistently identified a moderate number of significantly modulated pathways (5–10) across all contrasts (Figure 4A–C). CAMERA performed similarly but did not detect modulated pathways in the 1% O_2_, CyA+ vs 1% O_2_, CyA– contrast. PaintOmics and multiGSEA identified a single modulated pathway in the double-treatment contrast, i.e., 19% O_2_, CyA– vs 1% O_2_, CyA+. mBONITA is sensitive to modulated pathways in cases where the fold-changes are relatively low in all the omics data sets (1% O_2_, CyA+ vs 1% O_2_, CyA−), identifying 62 modulated pathways. In these cases, we encourage caution and interpretation of *p*-values in combination with mBONITA’s node modulation scores, which incorporate fold-changes, in order to select pathways for further study. Out of 13 key KEGG pathways known to be involved in the mechanism of the HIF1α-mediated chemotactic response of human B cells to O_2_ gradients and treatment with CyA (Supplementary Table S2),^14^ only mBONITA, ActivePathways and CAMERA identified pathways as being modulated. ActivePathways and CAMERA identified glycolysis as being modulated between samples grown at 19% O_2_ and 1% O_2_, regardless of CyA treatment, in line with known biology.^56^mBONITA identifies the upstream regulatory PI3K-Akt signaling pathway (reviewed in^56^) and the downstream effector, regulation of actin cytoskeleton,^57^ as modulated under these conditions (Figure 5D). These results suggest that mBONITA is more sensitive to moderate fold-changes across multiple data sets and returns specific results that are in line with the known biology of the condition under study.

## 4 Conclusion

The increasing availability of complementary high-throughput omics data sets profiling the same biological systems requires new computational tools for integrated analysis, especially pathway enrichment analysis. These integrated analyses identify modulated biological processes that are not apparent in either gene- or pathway-level analysis of individual data sets. Topology-based pathway analysis methods allow more thorough analysis of the overall modulation of signaling networks than gene set analysis.^7,8^ Here we present the algorithm **m**ultiomics **B**oolean **O**mics **N**etwork **I**nvariant-**T**ime **A**nalysis (mBONITA), which builds on our previously published methods^11,12^ to identify Boolean regulatory rules for known signaling network topologies, using a combined multiomics data set. We demonstrate the utility of mBONITA on a multiomics data set obtained from RAMOS B cells and show that this integrated analysis identifies modulated pathways that are not identified in single-data set analysis with other published methods. mBONITA also calculates a unique node modulation score that is complementary to gene-level expression analyses and, in our case study, uniquely identifies drivers of the underlying biological processes. Caveats of this method include (**1**) the need to appropriately normalize and combine multiomics data sets to minimize batch and scale effects, (**2**) the limited availability of prior-knowledge networks for pathway analysis, and (**3**) the possibly nonspecific inflation of *p*-values when observed fold-changes are small. These difficulties can easily be overcome with careful evaluation of data prior to the use of mBONITA and interpretation of the results, especially by simultaneously considering the node modulation scores and overall pathway modulation while selecting candidates for further study. This combination of gene-level and pathway-level metrics allows mBONITA to rigorously improve integrated analysis of multiomics data.

## Supporting Information Available

Supplementary Table S1: Experimental conditions in the three data sets from RAMOS B cells; Supplementary Table S2: KEGG pathways used to construct a network linking HIF1α and cytoskeletal changes; all pathways are *Homo sapiens*-specific; Supplementary Figure S1: Transcriptomics analysis of RAMOS B cells grown under three conditions; Supplementary Note S1: mBONITA’s node importance score reliably identifies highly influential nodes but cannot distinguish between similarly noninfluential nodes (PDF)
Supplementary File S1: Excel workbook containing tables of differentially expressed genes in GEO entry GSE212853 identified by DESeq2 and limma in all three contrasts; Sheets within this workbook are labeled with the method name (XLSX)
Supplementary File S2: Table of enriched pathways in the differentially expressed genes in GSE212853 identified by enrichr in all three contrasts (TXT)
Supplementary File S3: Table of modulated pathways in GSE212853 identified by mBONITA in all three contrasts (TXT)
Supplementary File S4: Table of modulated pathways in GSE212853 identified by ActivePathways in all three contrasts (TXT)
Supplementary File S5: Table of modulated pathways in GSE212853 identified by CAMERA in all three contrasts (TXT)
Supplementary File S6: Table of modulated pathways in GSE212853 identified by PaintOmics in all three contrasts (TXT)
Supplementary File S7: Table of modulated pathways in GSE212853 identified by multiGSEA in all three contrasts (TXT)
Supplementary File S8: Table of modulated pathways in GSE212853 identified by leapR in all three contrasts (TXT)
