# Supplementary material for: Executable Network Models of Integrated Multiomics Data
Source: J Proteome Res. 2023 Mar 31;22(5):1546–56. doi: 10.1021/acs.jproteome.2c00730 (PMC10167691; doi:10.1021/acs.jproteome.2c00730)
Supplement: Supplementary file 1 — pr2c00730_si_001.pdf [file pr2c00730_si_001.pdf]

Supplementary Information for "Executable network models of integrated multi-omics data"

Mukta G. Palshikar [†], Xiaojun Min [‡], Alexander Crystal [‡,¶], Jiayue Meng [‡], Shannon P. Hilchey [§], Martin S. Zand [§,‖] and Juilee Thakar [*,†,⊥,#]

†*Biophysics, Structural and Computational Biology Program, University of Rochester Medical Center, Rochester, NY*

‡*University of Rochester, Rochester, NY*

¶*Current affiliation: Khoury College of Computer Sciences, Northeastern University, Boston, MA*

§*Department of Medicine, Division of Nephrology, University of Rochester Medical Center, Rochester, NY*

‖*Clinical and Translational Science Institute, University of Rochester Medical Center, Rochester, NY*

⊥*Department of Microbiology and Immunology, University of Rochester Medical Center, Rochester, NY*

#*Department of Biostatistics and Computational Biology, University of Rochester Medical Center, Rochester, NY*

E-mail: Juilee_Thakar@URMC.rochester.edu

Supporting Information Available

List of Supplementary Materials

1. **Supplementary Tables**
   (a) Supplementary Table 1 Experimental conditions in the three datasets from RAMOS B cells.
   (b) Supplementary Table 2 KEGG Pathways Used to Construct a Network Linking HIF1α and Cytoskeletal Changes. All pathways are *Homo sapiens*-specific.
2. Supplementary Figure 1 Transcriptomics analysis of RAMOS B cells grown under three conditions
3. **Supplementary Files (available as separate xlsx or txt files):**
   (a) **Supplementary File 1, supplementary_file_1_mbonita.xlsx** Excel workbook containing tables of differentially expressed genes in GEO entry GSE212853 identified by DESeq2 and limma in all three contrasts. Sheets within this workbook are labeled with the method name.
   (b) **Supplementary File 2, supplementary_file_2_mbonita.txt:** Table of enriched pathways in the differentially expressed genes in GSE212853 identified by enrichr in all three contrasts.
   (c) **Supplementary File 3, supplementary_file_3_mbonita.txt:** Table of modulated pathways in GSE212853 identified by mBONITA in all three contrasts.
   (d) **Supplementary File 4, supplementary_file_4_mbonita.txt:** Table of modulated pathways in GSE212853 identified by ActivePathways in all three contrasts.
   (e) **Supplementary File 5, supplementary_file_5_mbonita.txt:** Table of modulated pathways in GSE212853 identified by CAMERA in all three contrasts.

(f) **Supplementary File 6, supplementary_file_6_mbonita.txt:** Table of modulated pathways in GSE212853 identified by PaintOmics in all three contrasts.

(g) **Supplementary File 7, supplementary_file_7_mbonita.txt:** Table of modulated pathways in GSE212853 identified by multiGSEA in all three contrasts.

(h) **Supplementary File 8, supplementary_file_8_mbonita.txt:** Table of modulated pathways in GSE212853 identified by leapR in all three contrasts.

(i) **Supplementary Note 1:** mBONITA's node importance score reliably identifies highly influential nodes but cannot distinguish between similarly non-influential nodes.

Supplementary Table 1: Experimental conditions in the three datasets from RAMOS B cells.

Data type	O_2	CyA	CXCL12
Proteomics	19%	−	−
	1%	−	−
	1%	+	−
Transcriptomics	19%	−	−
	19%	+	−
	1%	−	−
	1%	+	−
Phosphoproteomics	19%	+	+
	19%	−	+
	19%	+	−
	19%	−	−
	1%	+	+
	1%	−	+
	1%	+	−
	1%	−	−

Supplementary Table 2: KEGG Pathways Used to Construct a Network Linking HIF1α and Cytoskeletal Changes. All pathways are *Homo sapiens*-specific. Pathways highlighted with an asterisk (*) were used as starting networks in the experiment described in Supplementary Note 1 below.

MAPK signaling	hsa04010
Chemokine signaling	hsa04062
NF-kappa B signaling	hsa04064
HIF-1 signaling*	hsa04066
mTOR signaling	hsa04150
PI3K-Akt signaling*	hsa04151
VEGF signaling	hsa04370
Cell adhesion*	hsa04514
C-type lectin receptor signaling	hsa04625
JAK-STAT signaling	hsa04630
TNF signaling	hsa04668
Leukocyte transendothelial migration*	hsa04670
Regulation of actin cytoskeleton*	hsa04810

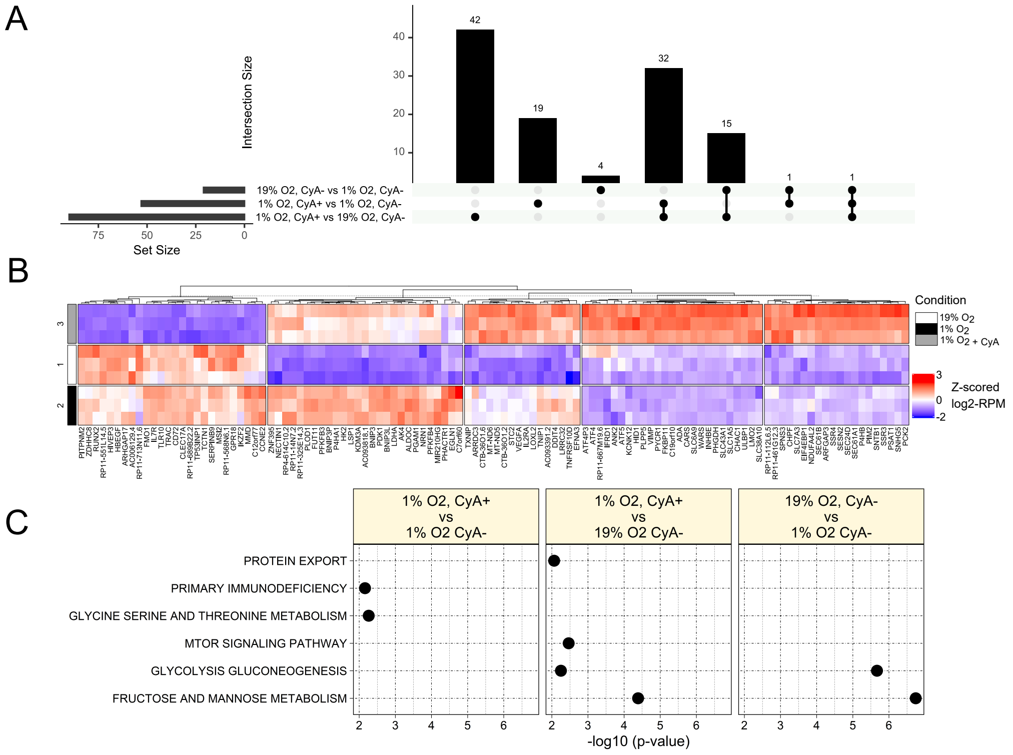

Supplementary Figure 1: Transcriptomics analysis of RAMOS B cells grown under three conditions. (A) Numbers of differentially expressed (DE) genes identified by DESeq2 in all three contrasts (absolute log_2-fold change > 0.5 and Bonferroni-adjusted $p < 0.05$) (B) z-scored log_2(RPM) values of DE genes identified in all contrasts. Experimental conditions are indicated by colors as shown in the legend. (C) Over-representation analysis of DE genes in all three contrasts (un-adjusted $p < 0.01$). Complete tables of DE genes and over-represented pathways may be found in Supplementary File 1 and Supplementary File 2 respectively.

Supplementary Note 1: mBONITA's node importance score reliably identifies highly influential nodes but cannot distinguish between non-influential nodes

mBONITA's node importance score identifies network nodes with high influence over signaling flow through the network due to their unique connectivity and location in the network topology. To confirm this dependence, we designed an experiment in which we randomized the network topology of real-life signaling networks and evaluated the effect of this randomization on the node importance scores calculated by mBONITA. We selected five KEGG networks (marked with asterisks in Supplementary Table 2). We used the double edge swap procedure implemented in the networkx Python package[1] to generate networks in which edges were swapped while maintaining degree distribution. The number of edge swaps was equal to half the number of original edges, i.e., efforts were made to keep the number of edges in the scrambled network as close to that in the original network as possible. Activation and inhibition signals (i.e., directionality) were assigned randomly as the edge swap procedure is only defined for undirected networks. 50 such networks were generated for each of the five starting networks. The mBONITA pipeline was used to calculate node importance scores for these scrambled networks and a distribution of importance scores was generated for each node. A p-value was calculated by comparing the node importance scores in these scrambled networks to those in the original network. Note that one distribution consisting of fifty node importance scores was generated for one node in one of the five original KEGG networks.

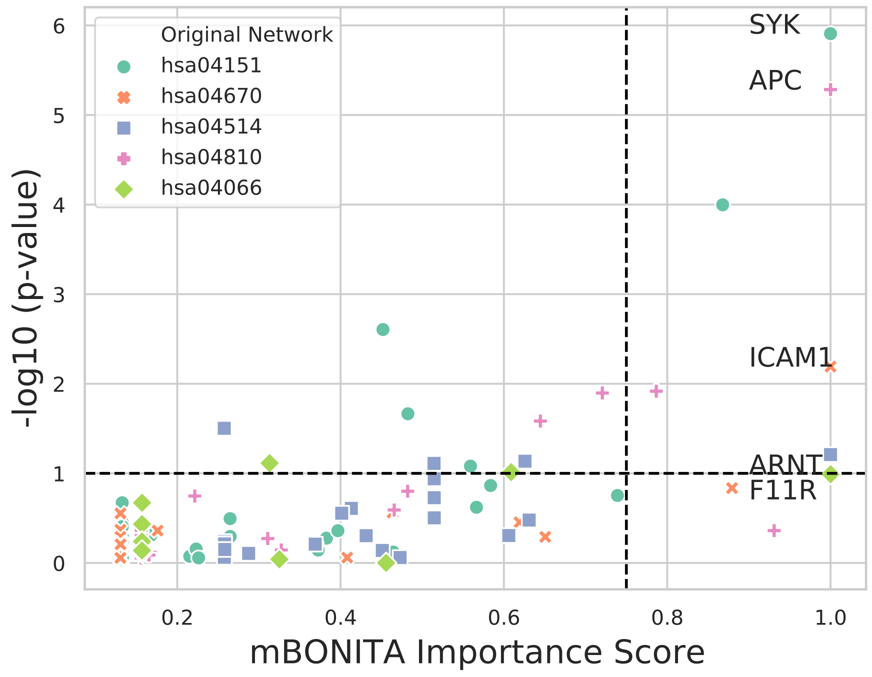

Supplementary Figure 2: **Impact of pathway topology on node importance score**. Relationship between mBONITA's node importance score and $-log_{10}p - value$ calculated by shuffling network topologies. Each point represents a gene in one of the original starting networks, which are indicated by distinct shapes and colors. Genes with an original node importance score $= 1$ are labeled with their names. Red dashed lines indicate the thresholds of $p = 0.1$ and importance score $= 0.75$.

All genes with the maximum importance scores of 1 in the original networks were found to be significant at $p < 0.1$ using this p-value calculation. However, most genes with importance scores below 1 were not assigned significantly different p-values in the shuffled networks (i.e, $p < 0.1$) (Supplementary Figure 2). This indicates that mBONITA's importance score reliably identifies high-importance nodes in signaling networks but it is less reliable at the lower end of the $[0, 1]$ scale, and cannot distinguish between nodes of low signaling influence. We caution the reader that this is a small simulation-based experiment with a limited set of starting networks, and these conclusions are speculatory in nature. We do not offer this p-value calculation as a part of the standard mBONITA workflow as it is time-consuming and adds little to the ranking of nodes by importance score.

References

(1) Hagberg, A. A.; Schult, D. A.; Swart, P. J. Exploring Network Structure, Dynamics, and Function using NetworkX. Proceedings of the 7th Python in Science Conference. Pasadena, CA USA, 2008; pp 11 – 15.
